# Gestation-Stage Related Changes in the IGF System Components in the Equine Placenta

**DOI:** 10.3390/biom15081135

**Published:** 2025-08-06

**Authors:** Kirsten E. Scoggin, Fatma Adlan, Carleigh E. Fedorka, Shimaa I. Rakha, Tom A. E. Stout, Mats H. T. Troedsson, Hossam El-Sheikh Ali

**Affiliations:** 1 Department of Veterinary Science, University of Kentucky, Lexington, KY 40546, USA; kirsten.scoggin@uky.edu (K.E.S.); taestout@uky.edu (T.A.E.S.); m.troedsson@uky.edu (M.H.T.T.); 2 Department of Theriogenology, Faculty of Veterinary Medicine, Mansoura University, Mansoura 35516, Egypt; fatmaadlan83@mans.edu.eg (F.A.); shimaa_ibrahim@mans.edu.eg (S.I.R.); 3 Department of Animal Sciences, Colorado State University, Fort Collins, CO 80523, USA; carleigh.fedorka@colostate.edu

**Keywords:** IGF system, VEGF, equine, chorioallantois, placenta

## Abstract

The insulin-like growth factor (IGF) system regulates implantation, placental development, and angiogenesis in eutherian mammals. However, little is known about the changes in this system in equine placenta (chorioallantois; CA) and the endometrium (EN) during pregnancy, or the relationship to vascular endothelial growth factor (*VEGF*) expression. The current study investigated the expression of the IGF system components, namely the ligands (*IGF1* and *IGF2*), their receptors (*IGF1R*, *IGF2R*, and *INSR*), and their binding proteins (*IGFBPs* and *IGF2BP*s) in equine CA at 45 days, 4, 6, 10, and 11 months of gestational age (GA) and immediately postpartum (PP), and in equine EN at 4, 6, 10, and 11 months GA. IGF1 immunolocalization and serum concentrations were also evaluated across gestation. *IGF1* mRNA expression in CA increased from day 45 to peak at 6 months and then gradually declined to reach a nadir in PP samples. This profile correlated positively with the *VEGF* expression profile (r = 0.62, *p* = 0.001). In contrast, *IGF2* expression in CA was not correlated with *VEGF* (*p* = 0.14). Interestingly, *IGF2* mRNA was more abundant in equine CA than *IGF1* (*p* < 0.05) throughout gestation. Among the IGFBPs investigated in CA, the expression of *IGFBP2* and *IGF2BP2* was highly abundant (*p* < 0.05) at day 45 compared to other GAs. Conversely, mRNA expression for *IGFBP3* and *IGFBP5* was more abundant (*p* < 0.05) in PP than at all investigated GAs. Immunohistochemistry revealed that IGF1 is localized in the equine chorionic epithelium (cytoplasm and nucleus). IGF1 serum concentrations peaked at 9 months and declined to their lowest levels PP. In conclusion, this study demonstrates a positive correlation between *IGF1* and *VEGF* expression in equine CA during gestation, suggesting that the IGF system plays a crucial role in placental angiogenesis by regulating *VEGF*.

## 1. Introduction

In mammals, the placenta is a transient organ that facilitates the exchange of gases, nutrients, and waste products between the maternal and fetal circulations during pregnancy. Additionally, it serves as a crucial source of pregnancy-related hormones and growth factors, which are essential for establishing and maintaining pregnancy [1]. Therefore, the successful implantation and development of the placental vasculature are crucial for normal fetal growth and development. Placental angiogenesis is regulated by various factors, including vascular endothelial growth factor (VEGF), a key player in placental vascular formation and development [2].

The insulin/insulin-like growth factor (IGF) system regulates fetal and placental growth and development. The IGF system regulates VEGF expression, impacting implantation and placental angiogenesis [3]. In addition, various components of the IGF system promote fetal development and growth [4]. The IGF system comprises ligands (IGF1 and IGF2), transmembrane receptors (IGF1R, IGF2R, and INSR (also known as IR; insulin receptor)), IGF binding proteins (IGFBP1–IGFBP10), and IGF2 mRNA-binding proteins (IGF2BP1–IGF2BP3) [4]. IGF1 and IGF2 are single-chain polypeptides homologous with insulin [5]. Both IGFs execute their biological functions primarily through binding to the IGF1R. IGF1 and IGF2 bind to IGF1R [6], inducing a receptor autophosphorylation that in turn leads to the activation of the PI3K and MAPK (ERK) pathways. This signaling cascade results in the amplified transcription of genes and increased biosynthesis of proteins involved in cell migration, proliferation, increased amino acid uptake, survival (downregulation of cellular apoptosis), and differentiation [5]. IGF2 binds with high affinity to INSR and IGF2R, whereas IGF1 has a much lower affinity for these two receptors [7]. IGF2R does not belong to the family of receptor tyrosine kinases [8]; hence, its downstream signaling cascade is different from that of IGF1R and INSR. IGF2R binds IGF1 and mannose-6-phosphate residues with high affinity [9], and its main role appears to be the clearance of extra IGF2 and mannose-6-phosphate-containing lysosomal enzymes [10]. Besides IGFRs, there are IGF-binding proteins that bind IGF molecules with high affinity. Some of the IGFBP family members possess an even higher affinity for IGF1 or IGF2 than IGFRs [11]. Therefore, circulating IGFBPs regulate the ligand’s access to receptors, thereby controlling bioavailability and acting as regulators of IGF activity.

The IGF system is species-specific, e.g., the expression of IGF ligands, receptors, and binding proteins varies greatly across species. There are only a few reports on equine mRNA and/or protein expression pertaining to the members of the IGF system. Moreover, these studies investigated only limited periods of pregnancy. For instance, Gibson et al. [12] described the expression of IGF1 and IGF2, IGF1R, IGF2R, INSR, and IGFBPs in equine conceptus and corresponding endometria in days 7–28 of gestation. In addition, *IGF2* mRNA expression has been reported in equine conceptus/fetus and placenta between 20 and 150 days of gestational age (GA) [13]. Arai et al. [14] reported that IGF1 is localized to the microcotyledons of the equine placenta at 130, 208, and 312 days of GA. These authors further suggested that IGF1 is involved in stimulating equine placental development [14] and highlighted the need to investigate the expression of IGFRs and IGFBPs in the equine placenta to better understand cell sensitivity to IGFs and the bioavailability of IGFs. Taking all the facts into account, we aimed to investigate the expression of all major IGF system components and the relationship between IGFs and VEGF in equine chorioallantois (CA) and endometrium (EN) throughout gestation, as well as immediately after parturition.

## 2. Materials and Methods

### 2.1. Animal Use and Tissue Collection

All animal treatments were approved and carried out in compliance with the University of Kentucky’s Institutional Animal Care and Use Committee (Protocols #2014-1215 and #2014-1341). A total of 24 reproductively healthy mares were used in this study. All mares (*Equus caballus*) were mixed-breed, weighed 250 to 600 kg, and between the ages of four and nine years old. The mares were maintained on a pasture with free access to grass hay. All mares were pasture-bred, and the pregnancy was confirmed by transrectal ultrasonography between 14 and 35 days of gestation [15]. GA was determined by conceptus vesicle size, embryonic, and allantoic development during the first ultrasound examination. Blood, CA, and EN samples were collected at 4, 6, 10, and 11 months (n = 4/GA). CA and EN samples recovered at 4 to 11 months of gestation were collected postmortem, as described elsewhere [16]. In brief, the entire uterus was recovered immediately after pentobarbital euthanasia, following the American Veterinary Medical Association guidelines for animal euthanasia [17]. The CA was gently separated from the EN and kept in the stabilization solution (RNAlater, #AM7021, Thermo Fisher Scientific, Waltham, MA, USA) until the RNA extraction. Full-thickness tissue samples were collected from the body of the placenta (CA) and preserved in neutral buffered formalin for 24 h before being fixed in methanol for immunohistochemistry (IHC).

The CA samples (n = 4) from day 45 GA were retrieved from conceptuses collected by uterine lavage as described previously [18]. The postpartum (PP) samples were collected from CA immediately after normal parturition (n = 4) [19,20]. CA from day 45 and PP were preserved in RNAlater (Thermo Fisher Scientific) for RNA extraction, and in neutral buffered formalin (*w*/*v*) for 24 h before being fixed in methanol for IHC. For this study, the PP time point refers to the term placenta collected immediately after foaling.

Blood was collected via jugular venipuncture at each particular GA and PP into a red-top tube (10 mL; Monoject; VWR, Radnor, PA, USA) and serum was separated by centrifugation at 1500× *g* for 10 min at 4 °C and then stored at −20 °C until use.

### 2.2. RNA Isolation and Sequencing

RNA was isolated from CA and EN samples using a RNeasy Mini Kit (#74104, Qiagen, Germantown, MD, USA), according to the manufacturer’s instructions and as previously described [21,22]. Bioanalyzer^®^ was used to determine RNA content, purity, and integrity (Agilent, Santa Clara, CA, USA). The absorbance ratio A_230_/A_260nm_ was over 1.8, whereas the ratio A_260_/A_280nm_ was over 2.0, and an RNA integrity number greater than 8.0 in all samples.

All CA and EN samples were subjected to RNA sequencing by Novogene America (Sacramento, CA, USA). A total of 1 µg RNA for each sample was used. Library was prepared using the TruSeq Stranded mRNA Sample Prep Kit from Illumina (San Diego, CA, USA), following the manufacturer’s instructions. The adapter for Read 1 was AGATCGGAAGAGCACACGTCTGAACTCCAGTCACNNNNNNATCTCGTATGCCGTCTTCTGCTTG, with NNNNNN signifying the index sequence. The Read 2 adapter was AGATCGGAAGAGCGTCGTGTAGGGAAAGAGTGTAGATCTCGGTGGTCGCCGTATCAT. All reads were quantified using qPCR. Sequencing was performed on a HiSeq 4000 from Illumina using a HiSeq 4000 sequencing kit (version 1) to generate 150 bp paired-end reads. We used bcl2fastq v2.17.1.14 Conversion Software from Illumina to generate and demultiplex FASTQ files. We have deposited all sequencing data in the NCBI Sequence Read Archive via the Gene Expression Omnibus (GEO) under GEO Series accession numbers: GSE136691 and GSE108279.

### 2.3. IGF1 and IGF1R IHC

The formalin-fixed CA samples were dehydrated, embedded in paraffin using routine methods, and sectioned at a thickness of 5 μm. For IHC of the tissue sections, we employed a recombinant rabbit anti-IGF1 (8W6E4) monoclonal antibody (1:50; #MA5-35060, Thermo Fisher Scientific) and a rabbit anti-IGF1R polyclonal antibody (1:100; #PA5-79444, Thermo Fisher Scientific). Before incubation with the primary antibody, the tissue sections were subjected to heat-induced epitope retrieval using an EDTA-based pH 9.0 epitope retrieval solution for 20 min (IGF1) or using a citrate-based pH 6.0 epitope retrieval solution for 20 min (IGF1R). Paraffin sections were processed with the Leica BOND-MAX system (Leica Microsystems, Buffalo Grove, IL, USA) as described elsewhere [23,24]. Negative controls were prepared using rabbit IgG (Supplementary Figure S1; Santa Cruz Biotechnology, Inc., Dallas, TX, USA). Slides were evaluated at 100× and 400× magnification.

### 2.4. IGF1/IGF2 Immunoassay

Serum concentrations of IGF1 and IGF2 at day 45 and 4, 6, 10, and 11 months, and PP were measured in duplicate using a human-specific multiple sandwich immunoassay based on flowmetric MILLIPLEX MAP technology (#HIGFMAG-52K, MilliporeSigma, Burlington, MA, USA). Assays for measuring equine IGF1 are no longer commercially available; hence the use of the human-specific kit [25]. IGF molecules were extracted from the serum using an acid ethanol solution (87.5% Ethanol and 0.25N HCl, diluted 1:60) as per the kit instructions, while calibration standards were prepared in assay buffer, as described [26]. The assay sensitivity for IGF1 was 19 pg/mL (Minimum Detectable Concentration (MinDC); Two Standard Deviations (2SDs); MinDC + 2SDs) with a standard curve range of 41–30,000 pg/mL. The mean intra-assay coefficient of variation was 0.28%. The assay sensitivity for IGF2 was 919 pg/mL (MinDC + 2SDs) with a standard curve range of 892–650,000 pg/mL. IGF2 could not be detected in the tested equine sera. It is unclear why equine IGF2 was not detected using the human-based kit; however, the immunogen sequence is proprietary and unavailable to the public. The recommended acid ethanol extraction is validated for human samples and may not be adequate to isolate IGF2 from equine samples.

### 2.5. Data Analysis

RNA-Seq reads were analyzed using a bioinformatics pipeline described elsewhere [24,27,28]. In brief, the reads were first trimmed for quality and adapters using TrimGalore 0.4.4. The reads were mapped to the EquCab3.0 horse genome using STAR 2.5.2b. Gene expression was quantified using Cufflinks 2.2.1 with the Ensembl annotation (Equus_caballus_Ensembl_95 gtf). Before subjecting them to data analysis, gene expression values were normalized by a global normalization method called transcript per million (TPM), as described previously [29] and validated in our lab [19]. Statistical analysis to determine Pearson correlation coefficients among IGF system transcripts in CA and EN was performed using GraphPad Prism version 10.0.0 for Windows (GraphPad Software, Boston, MA, USA). One-way analysis of variance was used to compare the expression levels of target genes in equine CA and EN across different gestation points, followed by Tukey’s Honestly Significant Difference post hoc test. Serum IGF1 levels across GAs were analyzed similarly. A *p*-value < 0.05 was considered statistically significant. Data are expressed as the mean ± standard error of the mean (SEM) unless otherwise stated.

## 3. Results

### 3.1. IGFs

**Figure 1:**
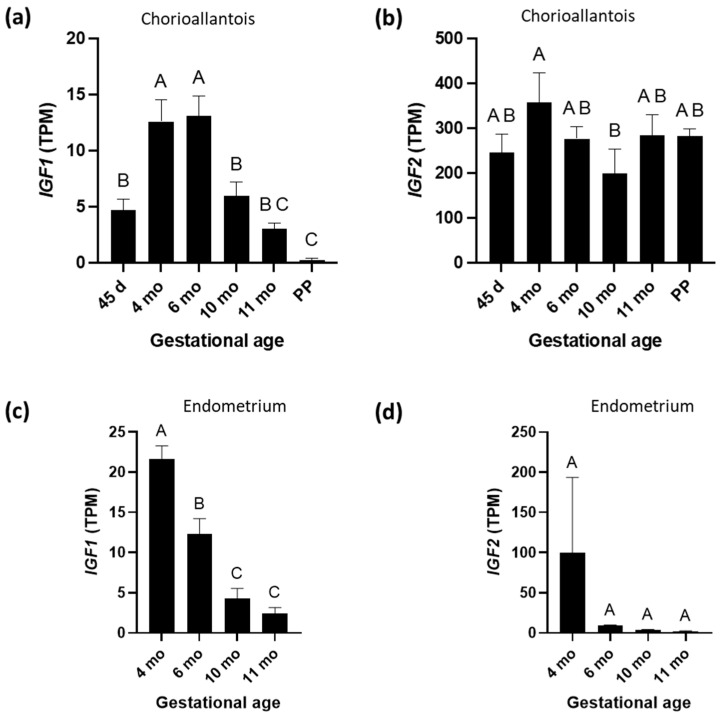
Insulin-like growth factor 1 (IGF1) and insulin-like growth factor 2 (IGF2) mRNA expression during equine pregnancy and immediately after parturition (postpartum (PP)) in chorioallantois and endometrium. (**a**) *IGF1* and (**b**) *IGF2* expression in chorioallantois. (**c**) *IGF1* and (**d**) *IGF2* expression in endometrium. TPM = transcripts per million. A, B, and C superscripts indicate significant differences between gestational ages (*p* < 0.05). Data are expressed as the mean ± standard error of the mean (SEM).

*IGF1* gene expression in CA started to rise from day 45 and peaked at 4–6 months, before a decline, with the lowest point occurring in PP (Figure 1a). Levels of mRNA encoding IGF2 were significantly greater than those encoding IGF1 in equine CA throughout gestation; however, the former showed less pronounced changes across GAs (Figure 1b). Gene expression for *IGF1* in the EN was highest at 4 months and decreased steadily to 11 months (Figure 1c).

**Figure 2:**
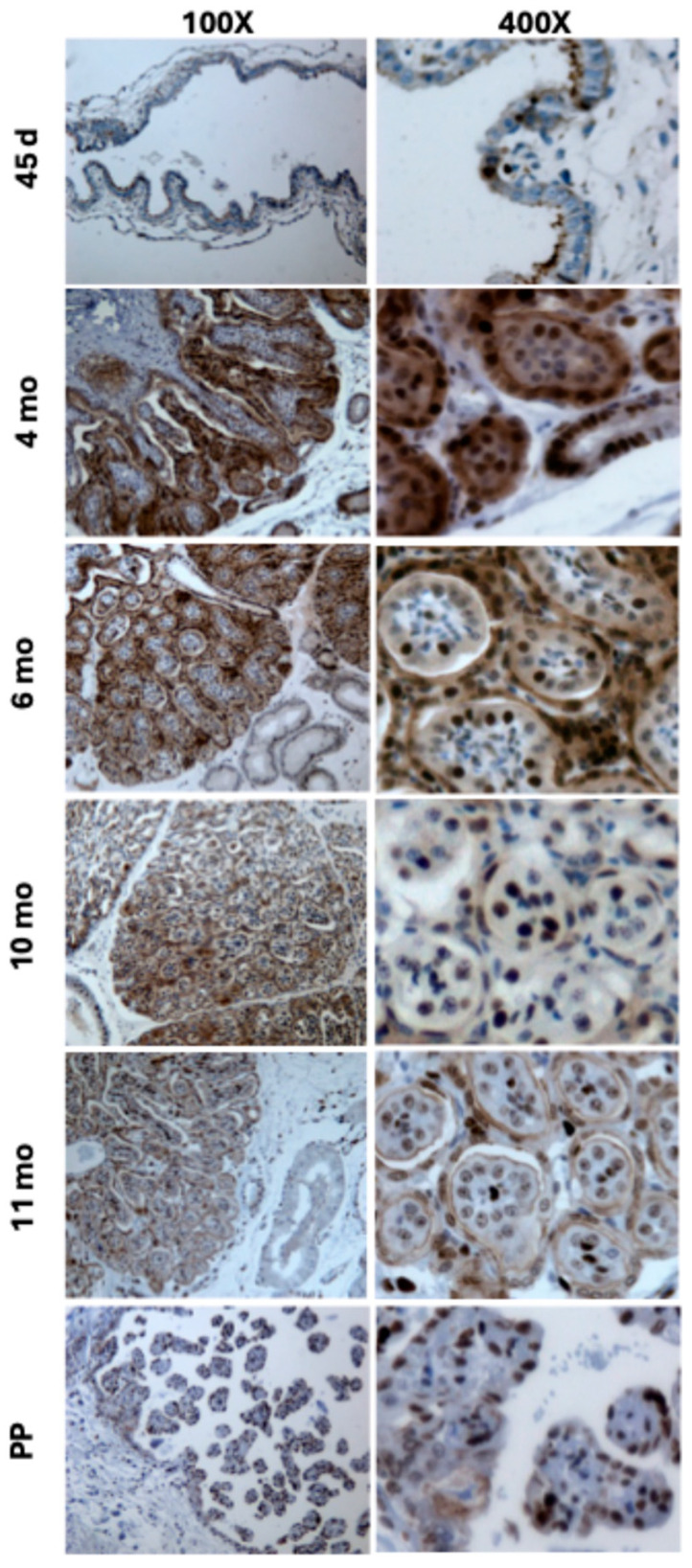
Representative photomicrographs of the equine chorioallantois stained for insulin-like growth factor 1 (IGF1) at day 45, and 4, 6, 10, and 11 months of gestation and immediately after parturition—postpartum (PP). Images shown at 100× (**left panels**) and 400× (**right panels**) magnification.

**Figure 3:**
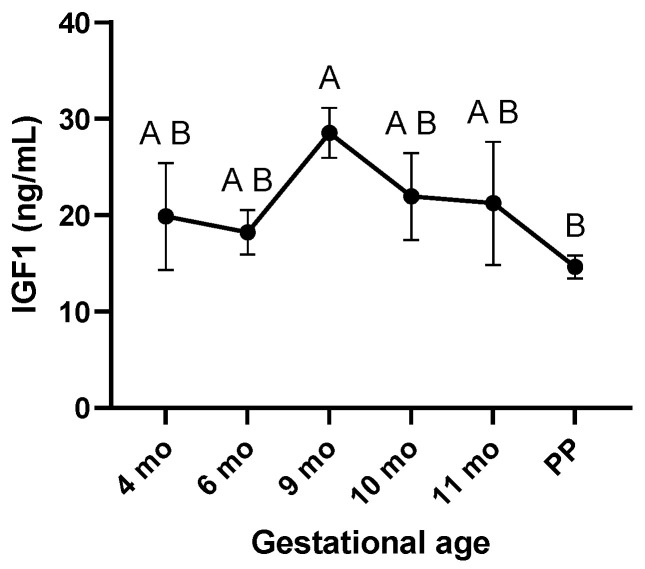
Peripheral insulin-like growth factor 1 (IGF1) concentrations throughout equine pregnancy. A and B superscripts indicate significant differences between gestational ages (*p* < 0.05). Data are expressed as the mean ± standard error of the mean (SEM).

The most intense immunostaining for IGF1 peptide was observed on the trophectodermal surface on day 45. Moreover, IGF1 detected at the chorionic epithelium at 4 and 6 months was mainly located in the cytoplasm, but towards the end of the pregnancy, IGF1 gradually translocated to the nucleus (Figure 2). The serum levels of IGF1 peaked at 9 months and gradually declined to reach a minimum at PP (Figure 3).

**Figure 4:**
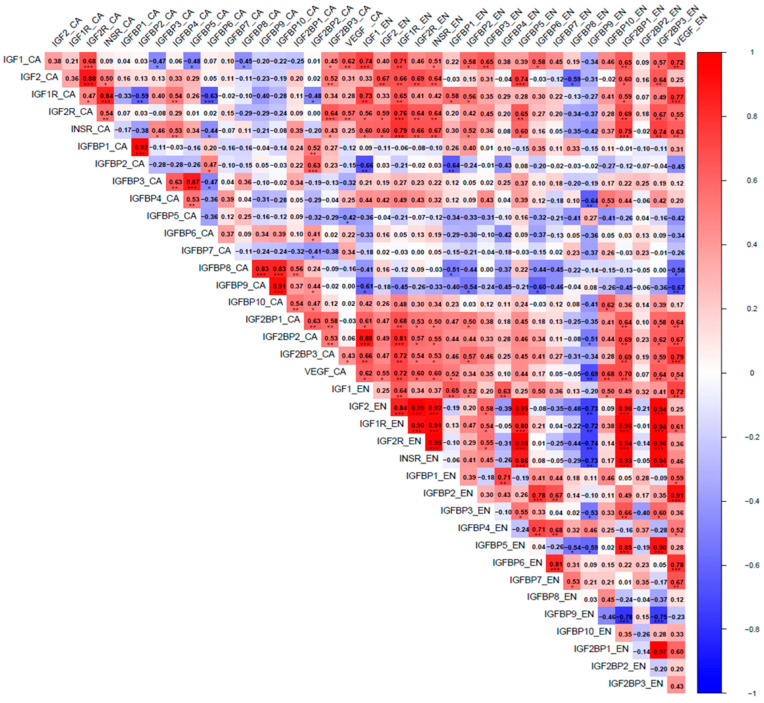
Pearson correlation coefficients heatmap among mRNAs encoding insulin-like growth factor 1 (IGF1), insulin-like growth factor 2 (IGF2), IGF1 receptor (IGF1R), IGF2 receptor (IGF2R), and insulin receptor (INSR), insulin-like growth factor-binding proteins (IGFBPs), insulin-like growth factor 2 binding proteins (IGF2BPs), and the vascular endothelial growth factor (VEGF) in equine chorioallantois (CA) and the endometrium (EN). The visualization was created using the corrplot package in R (version 2024.12.1+563), with significance levels denoted by asterisks (* *p* < 0.05, ** *p* < 0.01, *** *p* < 0.001). Correlations among mRNAs encoding the IGF system and the VEGF are colored from strong positive correlation (red) to no correlation (white), to negative correlation (blue).

The *IGF1* expression profile in CA was positively correlated with the *VEGF* expression in CA (r = 0.62, *p* < 0.01) and the EN (r = 0.72, *p* < 0.01), as shown in Figure 4 and Supplementary Figure S2 (Scoggin et al., 2025 [30]). The *IGF1* expression profile in the EN was positively correlated with the *VEGF* expression in the EN (r = 0.72, *p* < 0.01) (Figure 4; Supplementary Figure S2). On the other hand, the *IGF2* in CA and the EN was not correlated with the *VEGF* in CA or the EN (Figure 4).

### 3.2. IGFRs

**Figure 5:**
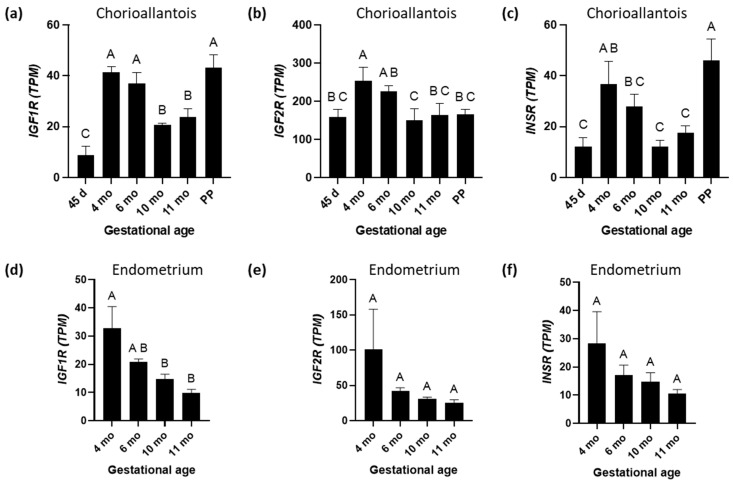
Insulin-like growth factor 1 receptor (IGF1R), insulin-like growth factor 2 receptor (IGF2R), and insulin receptor (INSR) mRNA expression during equine pregnancy and immediately after parturition (postpartum (PP)) in the chorioallantois and endometrium. (**a**) *IGF1R*; (**b**) *IGF2R*; and (**c**) *INSR* expression in chorioallantois. (**d**) *IGF1R*; (**e**) *IGF2R*; and (**f**) *INSR* expression in endometrium. TPM = transcripts per million. A, B, and C superscripts indicate significant differences between gestational ages (*p* < 0.05). Data are expressed as the mean ± standard error of the mean (SEM).

**Figure 6:**
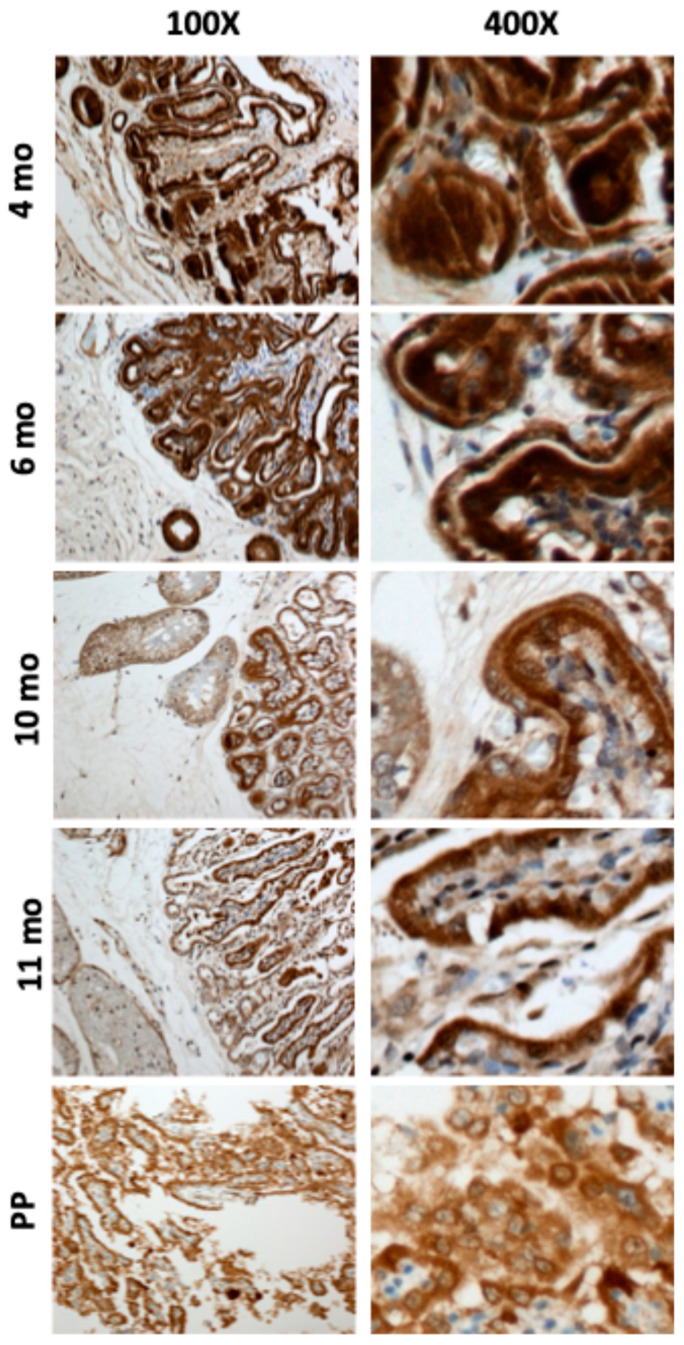
Representative photomicrographs of the equine chorioallantois stained for insulin-like growth factor 1 receptor (IGF1R) at 4, 6, 10, and 11 months of gestation and immediately after parturition—postpartum (PP). Images shown at 100× (**left panels**) and 400× (**right panels**) magnification.

The expression of *IGF1R* mRNA in CA increased from day 45 (*p* < 0.05) to higher levels at 4 and 6 months, then declined at 10 and 11 months before increasing again at PP (*p* < 0.05) (Figure 5a). A nearly identical expression pattern was observed for the *IR* (Figure 5c), and the similarity was supported by a strong positive correlation between gene expression for the two receptors (r = 0.84, *p* < 0.001). Expression of mRNA for the *IGF2R* increased from day 45 to 4 months (*p* < 0.05), then declined to reach a plateau at 10 months, 11 months, and PP (Figure 5b). We have observed a higher expression of mRNA encoding *IGF2R* than encoding *IGF1R* throughout gestation in equine CA (Figure 5a,b). *IGF2R* expression in CA was positively correlated with the expression of *IGF1* (r = 0.68, *p* < 0.001), *IGF2* (r = 0.88, *p* < 0.001), and *INSR* (r = 0.54, *p* < 0.01), as shown in Figure 4. In the EN, *IGF1R* expression was highest at 4 months and steadily decreased towards 11 months GA (Figure 5d). *IGF1* and *IGF2* in the EN positively correlated with *IGF1R* (r = 0.64 and 0.84, respectively). *IGF2*, but not *IGF1*, in the EN positively correlated with the *IGF2R* (r = 0.99, *p* < 0.001) and the *INSR* (r = 0.82) (Figure 4). Immunohistochemical analysis of IGF1R expression in equine CA revealed a distinct temporal and cellular staining pattern across gestation (Figure 6). IGF1R immunoreactivity was predominantly localized to the trophoblast epithelium, with staining observed in both the cytoplasm and along the cell membrane. At 4 and 6 months of gestation, IGF1R expression was relatively high and somewhat heterogeneous. By 10 and 11 months, staining became moderate and uniform across the trophoblastic layer. Minimal to no staining was observed in the mesenchymal core of the CA at any gestational stage.

### 3.3. IGFBPs

**Figure 7:**
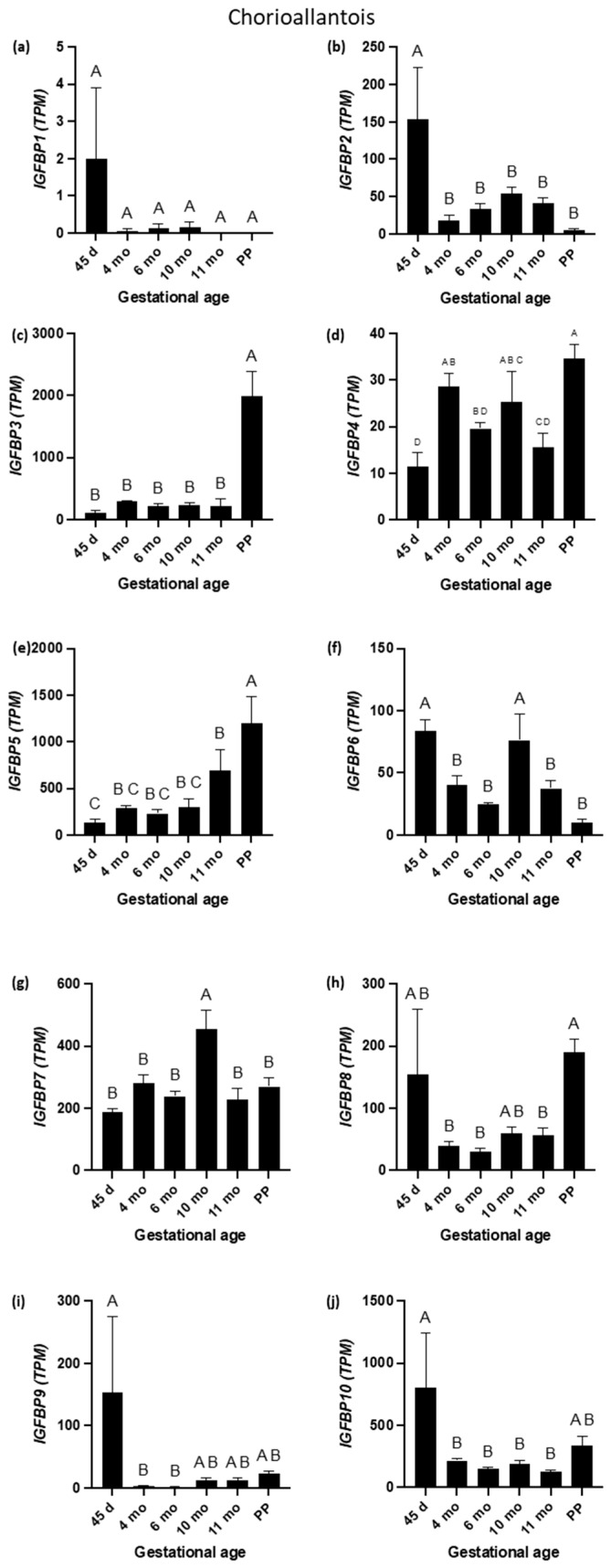
Insulin-like growth factor-binding protein 1 (IGFBP1) through to insulin-like growth factor-binding protein 10 (IGFBP10) mRNA expression during equine pregnancy and immediately after parturition (postpartum (PP)) in chorioallantois. (**a**) *IGFBP1*; (**b**) *IGFBP2*; (**c**) *IGFBP3*; (**d**) *IGFBP4*; (**e**) *IGFBP5*; (**f**) *IGFBP6*; (**g**) *IGFBP7*; (**h**) *IGFBP8*; (**i**) *IGFBP9*; and (**j**) *IGFBP10* expression in chorioallantois. A, B, C, and D superscripts indicate significant differences between gestational ages (*p* < 0.05). Data are expressed as the mean ± standard error of the mean (SEM).

**Figure 8:**
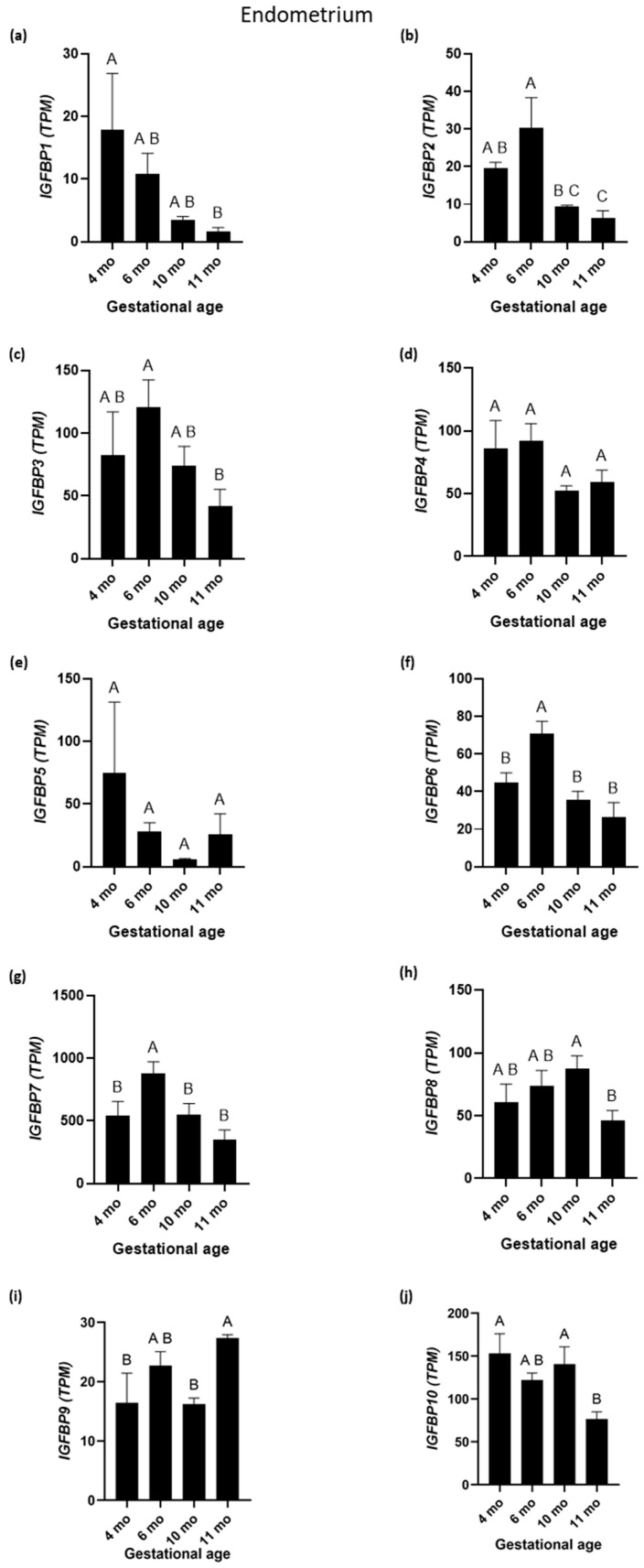
Insulin-like growth factor-binding protein 1 (IGFBP1) through to insulin-like growth factor-binding protein 10 (IGFBP10) expression during equine pregnancy in the endometrium. TPM = transcripts per million. (**a**) *IGFBP1*; (**b**) *IGFBP2*; (**c**) *IGFBP3*; (**d**) *IGFBP4*; (**e**) *IGFBP5*; (**f**) *IGFBP6*; (**g**) *IGFBP7*; (**h**) *IGFBP8*; (**i**) *IGFBP9*; and (**j**) *IGFBP10* expression in endometrium. A, B, and C superscripts indicate significant differences between gestational ages (*p* < 0.05). Data are expressed as the mean ± standard error of the mean (SEM).

**Figure 9:**
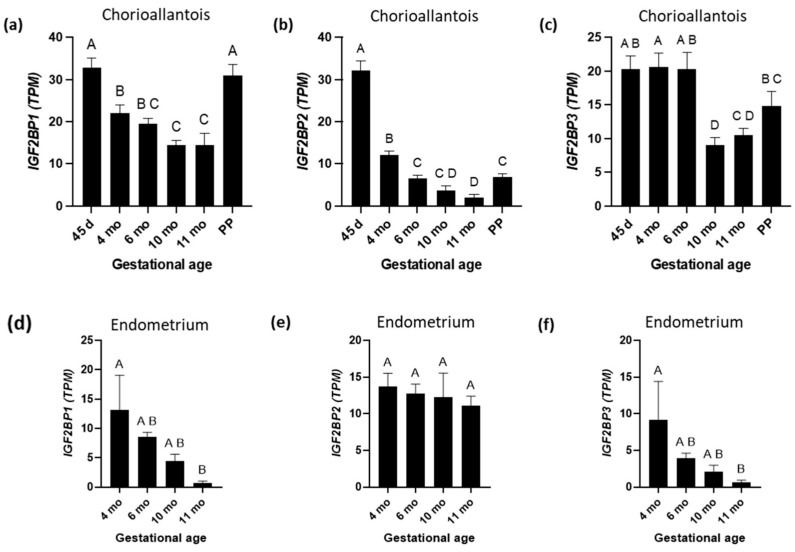
Insulin-like growth factor 2 binding protein 1 (IGF2BP1), insulin-like growth factor 2 binding protein 2 (IGF2BP2), and insulin-like growth factor 2 binding protein 3 (IGF2BP3) mRNA expression in equine placenta during pregnancy and immediately after parturition (postpartum (PP)) in the chorioallantois and endometrium. (**a**) *IGF2BP1*; (**b**) *IGF2BP2*; and (**c**) *IGF2BP3* expression in chorioallantois. (**d**) *IGF2BP1*; (**e**) *IGF2BP2*; and (**f**) *IGF2BP3* expression in endometrium. TPM = transcripts per million. A, B, C, and D superscripts indicate significant differences between gestational ages (*p* < 0.05). Data are expressed as the mean ± standard error of the mean (SEM).

The expression profiles of mRNAs encoding the IGFBP1 through 10 are depicted in Figure 7 (CA) and Figure 8 (EN). The expression profiles of mRNAs encoding IGF2-binding proteins are shown in Figure 9. Among the investigated *IGFBP*s, the expression levels of mRNAs encoding IGFBP2 and IGF2BP2 in CA were similar (r = 0.63, *p* < 0.001; Figure 4, Figure 7b and Figure 9b) with higher abundance at day 45 than at other GAs. By contrast, the levels of mRNAs for IGFBP3 and IGFBP5 in CA were higher at PP than at other GAs, and the two transcripts shared similar expression profiles (r = 0.87, *p* < 0.001; Figure 4 and Figure 7c,e). It is worth noting that the abundance of mRNA encoding IGFBP1 in CA samples was very low (except at day 25) (Figure 7a). In fact, 19 CA samples showed zero TPM. Four of the remaining five samples showed expression in the range of 0.21–0.59 TPM. In contrast, the expression of the *IGFBP1* was not low in the EN samples (Figure 8a). mRNAs for *IGFBP1* and IGFBP2 in CA were highly positively correlated (r = 0.92, *p* < 0.001). The *IGF1R* and *IGFBP6* in CA were negatively correlated (r = −0.63) (Figure 4).

The mRNA expression for the *IGFBP4* in CA fluctuated throughout pregnancy, with the highest expression reported at PP, which was significantly higher than at 45 days, 6 months, and 11 months (Figure 7d). The IGFBP1 and IGFBP2 exhibited similar expression patterns, with the highest levels occurring at day 45. The IGFBP3 and IGFBP5 showed identical expression patterns with a peak in term placenta. The IGFBP8, IGFBP9, and IGFBP10 showed maximum expression at day 45, followed by a decline in the latter part of gestation. Among the IGFBPs in CA, the IGFBP5 had the highest overall TPM values. The mRNA expression for the *IGFBP6* was significantly higher at 45 days and 10 months than at other GAs (Figure 7f) and was negatively correlated with the *IGF1R* (Figure 4). The mRNA expression for the *IGFBP7* was significantly higher at 10 months than at other GAs (Figure 7g). The mRNA expression for the *IGF2BP1* was significantly higher at 45 days and PP than at other GAs (Figure 9a). The mRNA expression for the *IGFBP8* was highest at PP, when it was significantly higher than at 4, 6, and 11 months (Figure 7h). *IGFBP9* and *IGFBP10* expression levels were highest at 45 days, which were significantly higher than at 4 and 6 months (for IGFBP9) or at 4, 6, 10, and 11 months (for IGFBP10), respectively (Figure 7i,j). The *IGFBP8* was positively correlated with the *IGFBP9* (r = 0.83, *p* < 0.001, Figure 4) and the *IGFBP10* (r = 0.83, *p* < 0.001, Figure 4). Additionally, *IGFBP9* and *IGFBP10* expression were positively correlated (r = 0.91, *p* < 0.001, Figure 4). In the EN, *IGFBP2*, *3*, *6*, and *7* exhibited similar expression patterns with a peak at 6 months, followed by a gradual decline to 11 months (Figure 8b,c,f,g). Of the IGFBPs expressed in the EN, the IGFBP7 had the highest overall TPM value. Of note, the expression of the *IGFBP5* in the EN was positively correlated with *IGF2* (r = 0.95), *IGF1*R (r = 0.80), *IGR2R* (r = 0.95), *IGFBP3* (r = 0.90), and *IGF2BP1* (r = 0.85) expression in the EN.

## 4. Discussion

The IGF system and the expression profile of its components in the embryo, placenta, and different reproductive tissues have been investigated in various species, including humans [31], water buffalo [32], sheep [33], pigs [34], and laboratory animals [35]. However, the IGF system of the horse placenta is much less studied, which may be due to difficulties linked to the diffuse, micro-cotyledonary epitheliochorial morphology of the equine placenta, which differs from other species’ placentas reported thus far [36]. Herein, we describe the expression profiles of a wide spectrum of IGF system components, namely IGF ligands, IGFRs, and IGFBPs in equine CA and EN at different gestational stages. Moreover, we report on the relationships between IGF system components and VEGF.

The genes for both the *IGF1* and *IGF2* are maternally imprinted (paternally expressed) in equine trophoblast [37]. Human and mouse models have shown that the regulation of imprinted gene expression can affect the implantation and growth of the fetus [38,39,40]. In the current study, IGF1 expression exhibited a unique profile, characterized by a peak at 4–6 months of gestation, followed by a gradual decline towards parturition (e.g., basal expression (nadir) at PP). The IGF1 expression profile was positively correlated with the mRNA expression of the *VEGF*, a well-known inducer of placental angiogenesis [41]. This positive relationship can be explained by the fact that the IGF1 is an upstream regulator of the VEGF [42,43]. In support, in vitro supplementation of the IGF1 is essential for the growth of human placental microvascular endothelial cells [44]. In mice, deletion of the *IGF1* gene results in fetal growth restriction and decreased placental size [45,46]. In newborn mice, knockout of the *IGF1* gene is also associated with a 30% growth reduction [47]. Administration of the IGF1 to guinea pigs during early to mid-gestation enhances late gestational fetal growth and survival close to term [34,48]. The positive correlation coefficient between the IGF1 and VEGF is concordant with previous reports on the increase in VEGF expression in the wall of subordinate follicles of mares’ ovaries after injection of the IGF1 [49,50]. Interestingly, the expression patterns of the *IGF1* and *VEGF* paralleled changes in estrogen levels in the blood or urine of mares during gestation. Estrogen concentrations rise to a peak at around the 7th month and then decline towards term [51,52,53]. In addition, estrogens stimulate *VEGF* expression and enhance the angiogenic activity of equine endothelial cells in vitro, underscoring estrogens’ importance in establishing the placental vascular network for optimal fetal development in the horse [54]; whether the stimulatory effect of estrogens and the IGF1 on VEGF expression is independent or related is not known. In the present study, we have demonstrated how the expression pattern of the *IGF1* transcript roughly parallels the IGF1 peptide; the latter was immunostained so its shift from intense and largely cytoplasmic presence at 4 and 6 months of gestation towards a nuclear presence in the later stages of pregnancy and PP was clearly visible. Upon binding to its receptor IGF1R, the IGF1 activates a signaling cascade involving a series of phosphorylation events on multiple downstream signaling proteins [55]. This cascade transmits the IGF1 signal toward the nucleus. Emerging evidence suggests that the IGF1-IGF1R complex itself may translocate to the nucleus following receptor activation, potentially carrying the IGF1 along. Therefore, the nuclear staining of IGF1 may reflect its presence in the complex with the IGF1R within the nucleus as part of this signaling process. The nuclear translocation of the IGF1 protein in late gestation may indicate a shift in its function, possibly involving cell proliferation and transcriptional regulation. In this respect, a study in human intestinal epithelial cells revealed that nuclear localization of IGF1 promoted cell proliferation [56]. Arai et al. [14] reported that IGF1 immunostaining was robust around Day 130, when placental growth and attachment were still in progress, but declined on Days 208 and 312 of pregnancy in horses when CA attachment to the entire EN would be complete. While mRNA expression was highest at 4 and 6 months, the circulatory IGF1 protein concentrations peaked at 9 months and declined to their lowest levels at PP, a profile similar to that described in Spanish purebred mares [57].

In the current study, we found a higher abundance of IGF2 transcript than IGF1 transcript in equine CA regardless of the GA. This finding corroborated that of Miese-Looy et al. [34], who reported 100-fold greater levels of IGF2 over IGF1 transcripts, although in porcine maternal and fetal tissues. Loux et al. [58] also reported that, at the transcript level, IGF2 is the most abundant growth factor in the CA at day 45. In human pregnancy, IGF2 enhances extravillous cytotrophoblast proliferation and inhibits apoptosis in an in vitro model of early pregnancy [59]. The importance of the IGF2 in fetal and placental growth and development is supported by the finding that reduced fetal size in IGF2 knockout mice is accompanied by changes in morphological features and a reduction in the size of the placentas in these animals [46,60], and that ablation of the IGF2 results in fetal and placental growth restriction [61]. These findings support the hypothesis that the IGF2 produced by the trophectoderm/chorion acts in an autocrine and/or paracrine fashion to drive general placental growth and development [13].

Previous studies in primates, rodents, and ruminants concluded that neither IGF1 nor IGF2 ligands cross the placental barrier, such that their effects must be produced locally through binding to their receptors (IGF1R and IGF2R) on placental cells [35]. The IGF1 attaches preferentially to the IGF1R, whereas the IGF2 binds preferentially to the IGF2R and to the IGF1R (although with a much lower affinity) [62,63]. IGF1R staining in the equine CA is most evident in the trophoblast epithelium, which forms the outer layer interfacing with maternal tissues, suggesting a role in fetal growth or placental maturation. The localization to trophoblasts aligns with the IGF1R’s known role in cell proliferation, nutrient transport regulation, and placental development [64]. Interestingly, mRNA for the *IGF2R,* a maternally expressed gene, was more abundant in equine CA and the EN than the *IGF1R* throughout gestation; a similar relationship was seen in human decidua [65]. The IGF2R does not have tyrosine kinase activity or an auto-phosphorylation site, and it is thought that the principle function of this receptor is to clear IGF2 from the circulation; this is supported by studies demonstrating that mice lacking the IGF2R have much greater birth weights than wild-type littermates [60,66], and highlights the role of the IGF2R in restricting fetal growth. Indeed, the deletion of IGF2R leads to placental and fetal overgrowth in mice [45]. In the present study, the expression of the IGF2R was positively correlated with the IGF1 and IGF2. It is widely accepted that the IGF2R regulates IGF2 bioavailability, thereby controlling fetal growth [12], potentially caused by high levels of IGF2 [67].

The IGFBP and IGF2BP families are highly conserved across vertebrates and act as critical modulators of various signaling pathways, rather than mere transporters of IGFs. IGFBPs are closely related proteins that bind IGF molecules with high-affinity (IGFBP1-IGFBP6) or low-affinity (IGFBP7-IGFBP10) [68]. IGFBP1-6 expression has been well characterized in humans, cattle, and horses; however, a detailed description of IGFBP7-10 expression throughout gestation is lacking. In the study presented herein, we could not detect *IGFBP1* transcripts in most CA samples, but in the EN we observed high levels of these. Our results are consistent with those of Han et al. [65], who found IGFBP1 expression primarily on the maternal side of the human placenta, where it interacted with the fetal IGF2 produced by the trophoblasts. IGFBP1, one of the least studied IGFBPs, has a unique property of adhering to the fibroblast extracellular matrix, where it can potentiate the actions of IGF1 and IGF2. It has been proposed that the primary function of the IGFBP1 is to regulate the transfer of IGFs between the EN and the uterine lumen. However, it has also been suggested to play a more controversial role in placental development by enhancing both basal and IGF2-induced extravillous trophoblast migration [69,70]. Phosphorylated IGFBP1 typically binds the IGF1 with high affinity and inhibits its activity. However, during pregnancy, it undergoes dephosphorylation and proteolysis, producing isoforms with lower affinity for the IGF, thereby increasing IGF bioavailability [71]. Such regulation is likely a key mechanism for promoting tissue growth at the maternal–fetal interface during pregnancy.

IGFBP2 and IGF2BP2 genes shared similar expression profiles in CA, with the highest abundance at day 45 of gestation. Levels of mRNA encoding the IGFBP2 in the EN rose at estrus and through early pregnancy (Day 28), suggesting that estrogen and/or the presence of the conceptus may boost its expression [12]. IGFBP2 mRNA expression increased in the equine conceptus membranes from day 7 to 28. During the implantation period, transcriptomic studies have revealed that several placental growth factors and IGF2BPs are overexpressed in human trophectoderm cells [72]. The IGF2BP2 has been reported to induce trophoblast cell invasion and migration, and its protein level is markedly reduced in severely preeclamptic placentas [73]. The IGFBP2 can also be shown to promote cell movement through integrin signaling [74].

In the present study, the levels of *IGFBP3* and *IGFBP5* transcripts in CA were highly correlated during pregnancy and at parturition (r = 0.87). Both genes were expressed at low levels throughout gestation and increased markedly in term placenta. The IGFBP3 and IGFBP5 are involved in key cellular processes, including apoptosis, cell migration, and adhesion, and play an important role in transporting and modulating the bioavailability of IGFs, crucial for fetal development and tissue remodeling during and after pregnancy [74]. The IGFBP3 enhances cell adhesion and survival through integrin interactions and can exert pro-apoptotic effects independently of IGF signaling [75]. The IGFBP3 plays a significant role during implantation in early pregnancy in the sheep (Days 13 to 15), after which its expression remains low [76]. During early pregnancy in the horse, the IGFBP3 has been detected in association with the blastocyst capsule and in uterine luminal fluid [77], is expressed by conceptus membranes, and is upregulated in the EN of early pregnant mares [78,79]. However, the function(s) of the IGFBP3 during early pregnancy in the horse are not known. In the human placenta, the IGFBP3 is known to modulate IGF activity and to be expressed at high levels by trophoblasts and fibroblasts in the villous stroma [80], as well as in the amniotic and chorionic membranes [65]. The IGFBP3 undergoes posttranslational cleavage during pregnancy by a disintegrin metalloproteinase, reducing its IGF-binding affinity and thereby increasing IGF bioavailability [81].

The IGFBP5 promotes cell adhesion and survival and regulates metabolic processes. It also participates in extracellular matrix interactions, binding to collagen, laminin, and fibronectin, facilitating IGF availability near cell surfaces, potentially supporting tissue remodeling associated with pregnancy and PP recovery [74,82]. The coordinated actions of the IGFBP3 and IGFBP5 highlight their multifaceted roles in regulating cellular behavior during pregnancy. In water buffalo, IGFBP3 expression in cotyledons decreases as gestation advances, whereas the IGFBP5 increases in early pregnancy and declines thereafter [32]. In pigs, high IGFBP3 expression is associated with arrest of conceptus trophoblast cells, suggesting a role in IGF sequestration, while IGFBP5 expression declines after implantation and remains low at Day 50 of gestation [34]. In sheep, IGFBP3 expression in cotyledons decreases as gestation progresses, and is concentrated around maternal blood vessels in the caruncle, where it has been postulated to regulate IGF transport [83]. IGFBP5 expression in sheep is primarily in the luminal epithelium and endometrial glands, with levels increasing as pregnancy progresses [84]. Therefore, the elevation of the IGFBP3 and IGFBP5 in term placenta likely reflects their combined functions in IGF transport, regulation of IGF bioavailability and signaling, and direct involvement in tissue remodeling at and immediately following parturition.

The IGFBP7 is less well characterized, and there are few studies available regarding its role during pregnancy. Nawathe et al. [85] identified elevated IGFBP7 expression (both mRNA and protein) in the placenta of small for GA neonates, with a significant correlation between IGFBP7 mRNA levels and birthweight. Their study was the first to detect the IGFBP7 in the human placenta in the context of fetal growth. IGFBP7 expression is reduced in the trophoblast cells and villous tissues from unexplained recurrent spontaneous abortion patients, suggesting that the IGFBP7 promotes trophoblast invasion, a crucial process for successful pregnancy [86]. In our study, *IGFBP7* expression was upregulated at 6 months of gestation in equine EN and 10 months in equine CA. The literature about IGFBPs 8–10 is limited, and the proteins are defined as IGFBP-related proteins belonging to the CCN (cyr61, ctgt, and Nov) protein family [87]. In equine EN, the expression of *IGFBP8* and *IGFBP10* declined between 10 and 11 months of gestation, whereas *IGFBP9* expression increased between these timepoints. Further research is required to understand the functional role of these binding proteins in the placental IGF system.

## 5. Conclusions

In conclusion, this study provides the first comprehensive analysis of the IGF system and its relationship with VEGF in the equine CA and EN throughout gestation to parturition. The expression patterns of IGF1 and IGF2, along with their receptors (*IGF1R*, *IGF2R*, *INSR*), binding proteins (*IGFBPs*), and mRNA binding proteins (*IGF2BPs*), suggest a dynamic regulatory role during placental development, with IGF1 showing significant correlations with VEGF expression, suggesting a critical involvement in implantation and placental angiogenesis. IGF1 protein expression gradually shifted from the cytoplasm to the nucleus in term placenta, suggesting that IGF1 nuclear translocation may play a role in parturition. The findings underscore the importance of the IGF system in supporting fetal growth and placental function, highlighting potential avenues for further research on the mechanisms governing equine pregnancies.

## Data Availability

The original contributions presented in this study are included in the article. Further inquiries can be directed to the corresponding author.

## Supplementary Materials

The following supporting information can be downloaded at: https://www.mdpi.com/article/10.3390/biom15081135/s1, Figure S1: Representative photomicrograph of the equine chorioallantois stained for normal rabbit IgG at 6 months of gestation, serving as the negative control. Figure S2: Insulin-like growth factor 1 (IGF1) and vascular endothelial growth factor (VEGF) mRNA expression during equine pregnancy and immediately after parturition (postpartum (PP)).

## Abbreviations

The following abbreviations are used in this manuscript:

IGF	Insulin-like growth factor (e.g., IGF1 and IGF2)
VEGF	Vascular endothelial growth factor
CA	Chorioallantois
EN	Endometrium
GA	Gestational age
PP	Postpartum
IGF1R	Insulin-like growth factor 1 receptor
IGF2R	Insulin-like growth factor 2 receptor
INSR	Insulin receptor
IGFBPs	Insulin-like growth factor-binding proteins (e.g., IGFBP1-IGFBP10)
IGF2BPs	Insulin-like growth factor 2 mRNA-binding proteins (e.g., IGF2BP1-IGF2BP3)
TPM	Transcripts per million
SEM	Standard error of the mean
IHC	Immunohistochemistry
RNA-Seq	RNA sequencing
PI3K	Phosphoinositide 3-kinase
MAPK (ERK)	Mitogen-activated protein kinase (extracellular signal-regulated kinase)
MinDC	Minimum detectable concentration
2SDs	Two standard deviations
