# Supplementary material for: Gestation-Stage Related Changes in the IGF System Components in the Equine Placenta
Source: Biomolecules. 2025 Aug 6;15(8):1135. doi: 10.3390/biom15081135 (PMC12384007; doi:10.3390/biom15081135)
Supplement: Supplementary file 1 [file biomolecules-15-01135-s001.zip › biomolecules-3737929-supplementary.pdf]

Supplementary Materials

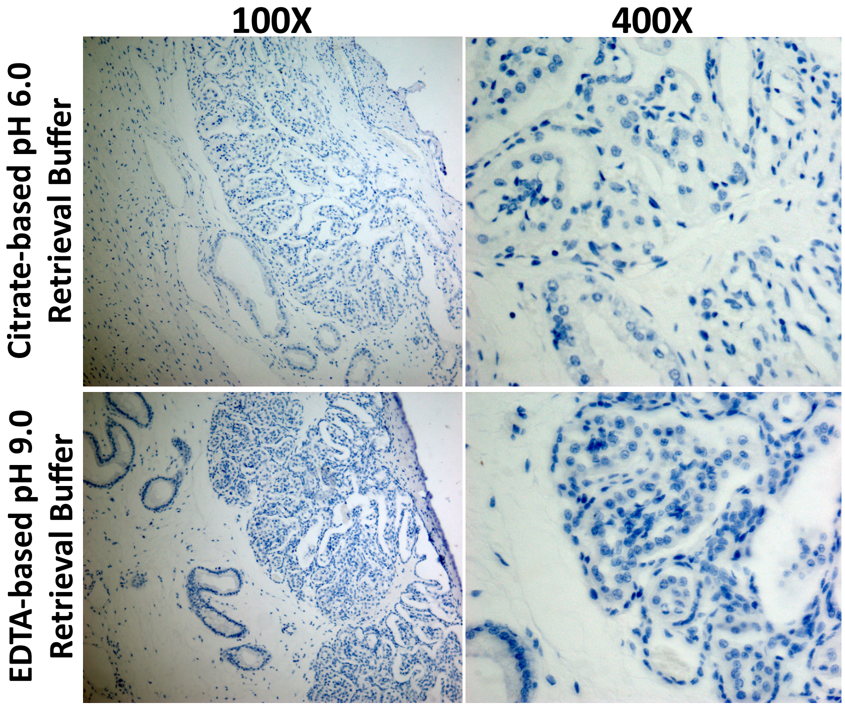

Figure S1: Representative photomicrograph of the equine chorioallantois stained for normal rabbit IgG at 6 months of gestation, serving as the negative control. The top panel is processed using a citrate-based pH 6.0 epitope retrieval solution for 20 min, and the bottom panel is processed using an EDTA-based pH 9.0 epitope retrieval solution for 20 min. Images shown at 100× (left panel) and 400× (right panel) magnification.

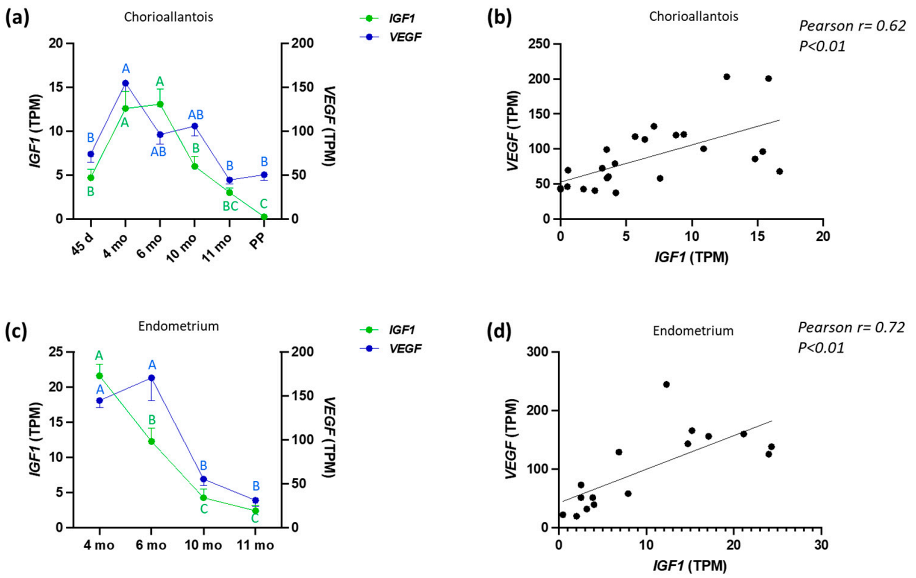

Figure S2. Insulin-like growth factor 1 (IGF1) and vascular endothelial growth factor (VEGF) mRNA expression during equine pregnancy and immediately after parturition (postpartum (PP)). (**a**) The expression of *IGF1* and *VEGF* in chorioallantois. (**b**) Pearson correlation coefficient (r = 0.62, $p < 0.01$) between the IGF1 and VEGF in the chorioallantois. (**c**) The expression of the *IGF1* and *VEGF* in the endometrium. (**d**) Pearson correlation coefficient (r = 0.72, $p < 0.01$) between the IGF1 and VEGF in the endometrium. TPM = transcripts per million. A, B, and C superscripts indicate significant differences between gestational ages ($p < 0.05$). Data are expressed as the mean ± standard error of the mean (SEM).
